# Analgesic effects of dexmedetomidine and remifentanil on periprocedural pain during percutaneous ablation of renal carcinoma

**DOI:** 10.1080/03009734.2020.1720047

**Published:** 2020-02-18

**Authors:** Egidijus Semenas, Maria Lönnemark, Pär Dahlman, Michael Hultström, Mats Eriksson

**Affiliations:** a Section of Anesthesiology and Intensive Care, Department of Surgical Sciences, Uppsala University, Uppsala, Sweden;; b Section of Radiology, Department of Surgical Sciences, Uppsala University, Uppsala, Sweden;; c Section for Integrative Physiology, Department of Medical Cell Biology, Uppsala University, Uppsala, Sweden

**Keywords:** Analgesia, dexmedetomidine, microwave ablation, radiofrequency ablation, remifentanil, renal carcinoma

## Abstract

**Background:** Percutaneous ablation of renal carcinoma is frequently a favourable treatment alternative, especially in elderly patients suffering from co-morbidities. Also, it is less resource-demanding than conventional surgery of renal carcinoma, and one may, therefore, assume that the incidence of this procedure may increase. Analgesia is necessary during this intervention. The aim of this study was to explore the possibility of analgosedation and its relation to patient comfort and safety during percutaneous ablation of renal carcinoma.

**Methods:** Forty-six patients, sedated with dexmedetomidine and remifentanil, supplemented with infiltration anaesthesia (lidocaine 1%), underwent percutaneous (radiofrequency or microwave) ablation of renal carcinoma in this prospective study.

**Results:** The patients expected pain intensity around the numerical rating score (NRS) 4.5 (interquartile range [IQR] 3.5–5.5), which was slightly lower than pain experienced during the procedure NRS 5 (IQR 2–7; *p* = 0.49). Eight percent of the patients needed supplementary morphine during the ablation procedure. Sedation score did not differ during ablation, at arrival to or discharge from the recovery ward. Median periprocedural treatment time was 12 minutes (IQR 12–16). Treatment time did not correlate with experienced pain (*R^2^*=0.000074, *p* = 0.96). The median length of stay in the recovery room was 120 minutes (IQR 84–154). There were seven serious adverse events.

**Conclusions:** This proof-of-concept study has shown that analgosedation during percutaneous ablation of renal carcinoma can be performed with a generally tolerable degree of patient satisfaction. However, pain occurs and should be managed adequately. Patient safety must be a major concern for the anaesthetic care.

## Introduction

The number of discovered cases of renal cancer is increasing, the most rapid increase being in small tumours (1). This seems to be due to more widespread imaging, where cross-sectional imaging techniques are used. As a consequence of earlier diagnosis, in combination with new treatment options, mortality in renal cancer seems to be decreasing (2). Optimal treatment may be dependent on both the type and the stage of renal cancer (3).

Advantages of radiofrequency ablation include the rare occurrence of deterioration of renal function with an overall 5-year survival rate of 58–85%, according to meta-analyses (4). Radiofrequency ablation is indicated in T1 tumours (i.e. the cancer cells are only growing in the most superficial layer of tissue, without growing into deeper tissues) in patients with advanced age and significant co-morbidities, including reduced renal function as well as single kidney. Major complication rates (e.g. relevant deterioration of renal function) are reported in 0–14% of interventions (4).

In a retrospective study, 19 patients with renal cell carcinoma were treated with radiofrequency ablation, while 21 patients were treated with partial nephrectomy. Length of hospital stay, mean procedural time, and blood loss were lower in patients treated with radiofrequency ablation. Elevated serum creatinine concentrations were noted in patients undergoing partial nephrectomy, but not in those undergoing radiofrequency ablation. Regarding the occurrence of metastasis, there were no differences (5,6).

Since radiofrequency ablations are performed in the radiological department, the anaesthetic procedure is more time-consuming and space-occupying than in a regular operating room, designed for pre- and postanesthetic management. Also, the distance between the radiological department and the surgical unit may cause problems when assistance is needed in an anaesthetic emergency. Furthermore, there is an advantage to the radiologist if the patient is cooperative and able to follow instructions. Thus, we decided to evaluate whether sedation in combination with local anaesthetic could be a favourable approach. Initially, we used midazolam in combination with remifentanil for this purpose. However, we had some complications in the form of mental confusion, too heavy sedation, agitation, etc.

Therefore, we decided to change this concept and instead use dexmedetomidine and remifentanil during percutaneous ablation of renal cell carcinoma. This drug combination has proven efficacy in several surgical procedures, including awake craniotomy (7). Initially, this regimen was, because of safety concerns, always commenced in the presence of both an experienced anaesthesiologist and a well-educated anaesthetic nurse. As we found this routine satisfactory, we decided to evaluate this procedure in a proof-of-concept study.

The primary aim of our study was to evaluate overall patient comfort, focussing on the possibility to substitute anaesthesia with analgosedation during percutaneous ablation of renal carcinoma.

The secondary aims were to:

- Evaluate anaesthetic consumption as well as analgesic requirements during the first 24 hours after the procedure.
- Evaluate patient safety during the per- and postprocedural phases of percutaneous ablation of renal cell carcinoma in analgosedated patients.
- Describe patient characteristics and periprocedural circumstances for reference and guidance in similar interventions, either therapeutic or diagnostic.

## Materials and methods

Fifty-one patients planned for percutaneous ablation of renal carcinoma were screened for participation in this study. All patients gave their oral and written consent to participate in this study, which was approved (2013/409) by the Regional Ethical Review Board at Uppsala University, Sweden. According to Eudralex, we do not see this study as a clinical trial, which would need approval by the Swedish Medical Products Agency. This study was registered at Clinicaltrials.gov on December 13, 2013.

In two patients, general anaesthesia was considered a better option, and one patient refused to participate in this study. Two patients were excluded because of other treatment options.

Demographic data of the 46 (22 female) remaining patients were as follows: age (mean ± SD) was 66 ± 12 years; mean weight 79 ± 16 kg; mean height 170 ± 9 cm. The majority of patients (37/46, 80%) had limited co-morbidities and were considered to be classified as ASA 2, according to the American Society of Anaesthesiologists (ASA) physical status classification system; some were classified as ASA 1 (*n* = 7), and only 2 were ASA 3.

Inclusion criteria were:

- Patients of both sexes, aged 18–80 years, able and willing to participate in this study.
- Renal cancer for which radiofrequency or microwave ablation was planned.
- Signed informed consent form.

Exclusion criteria were:

- Patient refusal.
- Pregnancy.
- Known allergy to dexmedetomidine and remifentanil.
- Atrioventricular block grade II or III or other significant cardiac conduction dysfunction.
- History of stroke.
- Low blood pressure not responding to treatment.

### Protocol

Before the onset of the procedure, patients were asked to assess expected maximal pain intensity during the radiofrequency/microwave ablation, according to a 11-point numerical rating scale (NRS), where ‘0’ is no pain and ‘10’ is the worst imaginable pain (8). The patients were informed that pain should be expected during the procedure and that analgesic drugs would be given on demand. In order to avoid bias due to expected different procedural pain levels (radiofrequency versus microwave ablation), all patients received identical information. Ethical considerations were based on our clinical experience from radiofrequency/microwave ablation and included an algorithm aiming to avoid unnecessary pain. During the postprocedural period in the recovery ward, patients were asked, approximately 15 min after arrival, to arbitrarily assess maximal pain intensity during the ablation and also to assess overall satisfaction, as a ‘satisfaction score’, with the sedation technique (1, dissatisfied; to 5, satisfied). Since the patients were transferred from the roentgenological department to the recovery ward, a time period of 30–45 min between discontinuation of dexmedetomidine and remifentanil and first estimation of pain was realistic. When leaving the recovery ward, the patients were, once again, asked to assess the overall satisfaction with the sedation technique and also to assess the maximal pain intensity during the recovery phase. Sedation during the percutaneous ablation procedure, at arrival to the recovery ward, and at discharge, was evaluated according to the Ramsey Sedation Scale Score (9), which describes a patient as follows:

1. anxious and agitated, or restless, or both
2. co-operative, oriented, and calm
3. responsive to commands only
4. exhibiting brisk response to light glabellar tap or loud auditory stimulus
5. exhibiting a sluggish response to light glabellar tap or loud auditory stimulus
6. unresponsive

Nineteen patients asking for premedication received paracetamol 1 g orally, approximately 1 h before start of the ablation procedure. No other analgesic was given before the radiofrequency ablation. Conventional anaesthetic variables (five-lead electrocardiogram, non-invasive blood pressure, heart rate, transcutaneous oxygen saturation, end-tidal carbon dioxide) were monitored. After administration of 0.5 mg of atropine, all patients received a continuous infusion of dexmedetomidine, starting at 0.4 μg kg^−1^ h^−1^ and remifentanil administered by a target-controlled infusion, where the initial plasma level was set to be 0.5 ng mL^−1^. When necessary, infusion rate of remifentanil was changed at the investigators’ discretion, in order to achieve a comfortable sedation for the patient. Dexmedetomidine infusion was kept stable throughout the procedure. Local anaesthetic (8–10 ml of lidocaine 1%) was infiltrated in the kidney at the radiologist’s discretion during the treatment. An anaesthetic nurse and/or anaesthetist was present during the entire procedure.

Serious adverse events (SAEs) were defined as complications that were either fatal, life-threatening, requiring hospitalization, resulting in persistent significant disability, or requiring intervention to prevent permanent damage. 

### Percutaneous ablation procedure

The procedure was performed under CT-guidance (Somatom Definition Flash, Siemens, Forchheim, Germany). In 42 sessions one tumour was ablated, and in 4 sessions two tumours were ablated. When an optimal needle path was identified, the skin and needle track were infiltrated with 8–10 ml local anaesthesia. Prior to ablation, core biopsies with a 1.2 mm cutting needle were taken (ProMag Ultra, Argon, Angiotech, Gainesville, FL, USA; or Quick Core Biopsy Needle, Cook Medical, Bloomington, IN, USA). Tumours were ablated using either a radiofrequency (RF) ablation system (Cool-tip RF Ablation system E Series, Medtronic, Boulder, CO, USA; *n* = 37) or two different microwave (MW) ablation systems (Emprint Ablation System Medtronic, Boulder, CO, USA, *n* = 5; or MicroThermeX, BSD Medical, Bountiful, UT, USA, *n* = 4). The choice of electrodes or probes was based on the manufacturers’ recommendations. Hydrodissection was used in 27 patients.

### Statistics

After validation and, when needed, confirmation of data was obtained from patients and co-workers in this study, the data were entered into an Excel spread sheet, serving as a database, from which calculations were performed. Values are, as considered appropriate, expressed as mean ± standard deviation (SD) and/or median, first (Q1) and third (Q3) quartiles, respectively. Paired Wilcoxon signed rank test was used for evaluating possible differences in expected versus periprocedural pain perception. A 40-patient sample size was calculated to achieve 85% power for the paired Wilcoxon signed rank test of expected versus experienced pain to detect a 2 NRS mean difference at the 5% significance level. *p* < 0.05 was considered significant.

## Results

### Tumoral growth

In 16 patients the renal carcinoma was exophytic, in 13 patients the tumour was endophytic, and in 17 cases it was both exophytic and endophytic. Three patients had bilateral renal tumours, and in two cases a suprarenal gland was involved. In one patient the procedure was interrupted because of severe pain.

### Expected and experienced pain

The patients expected pain around NRS 4–5 (mean: 4.6 ± 1.9; median 4.5, IQR 3.5–5.5), which was slightly lower than how they assessed their pain during the procedure (mean: 5.1 ± 2.8; median 5, IQR 2–7), although the difference was not statistically significant by Mann–Whitney (*p* = 0.49). Four patients (8%) needed supplementary morphine during the roentgenological interventional procedure.

### Anaesthetic consumption and effect

The average total dexmedetomidine given was 55 ± 20 µg, and remifentanil 371 ± 288 µg with an average maximal target concentration on target-controlled infusion for remifentanil of 2.92 ± 1.15 µg. This corresponded to 0.7 ± 0.2 µg kg^−1^ dexmedetomidine, and 4.8 ± 3.4 µg kg^−1^ remifentanil. Or, taking treatment time into account, dexmedetomidine 0.06 ± 0.038 µg kg^−1^ h^−1^ and remifentanil 0.37 ± 0.28 µg kg^−1^ h^−1^.

### Treatment time

**Figure 1.**
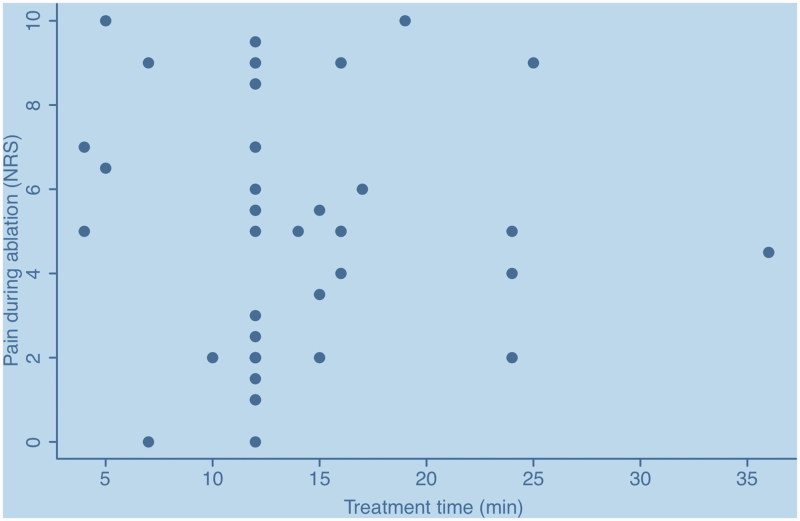
Treatment time did not correlate with experienced pain (*R^2^*=0.00007398, *p* = 0.9553).

Treatment time varied between 4 and 36 min. Average treatment time was 14.13 ± 6.7 min, median 12 (IQR 12–16) min. Treatment time did not correlate with experienced pain (*R^2^* = 0.00007398, *p* = 0.9553) (Figure 1).

### Treatment options and pain

**Figure 2.**
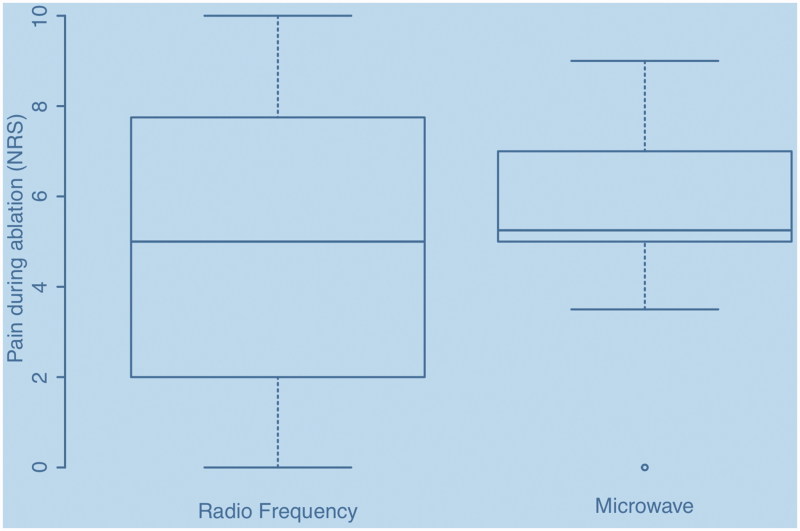
Pain median radio frequency ablation (5, IQR 2–7.4) versus microwave ablation (5, IQR 4.25–6.75).

Treatment options were either RF or MW. The majority, 37 patients, received RF, while 9 patients received MW. There was no difference between the two treatment groups in experienced pain level, average RF = 4.94 (SD 3.04) versus MW = 5.36 (SD 2.58) (Figure 2).

### Recovery

Maximal pain during the recovery phase was lower than both expected and experienced pain during the periprocedural phase (mean: 2.9 ± 2.5; median: 2.5, IQR 0–5). Sedation score (8) during the percutaneous ablation, at arrival to the recovery ward and at discharge from the recovery ward, did not differ (all occasions: median: 2, IQR 2–2).

Four patients (8%) experienced postoperative nausea and vomiting, which needed treatment with metoclopramide, ondansetron, betamethasone, or a mixture thereof, during the post-interventional phase. Thirty-five patients (73%) experienced pain (NRS > 0) during the recovery phase. Morphine, usually at low dose (mean: 3.5 mg; median: 1 mg; IQR: 0–7) was administered to 25 of these patients. Ketobemidone at 2 mg was given to 2 of these 35 patients. Paracetamol was given to 18 patients, who had not received paracetamol as premedication. Eleven patients were given clonidine intravenously. Fifteen patients needed mixtures of these analgesic drugs during the recovery phase.

Three patients needed supplementary morphine in the ward, and 15 of the 46 patients were given further paracetamol in the ward. One patient received ibuprofen for pain relief in the ward.

Mean length of stay in the recovery ward was 143 ± 168 min (median: 120 min; IQR: 84–154). One patient (see below) stayed overnight.

Mean satisfaction score was 3.7 ± 0.7 (median satisfaction score was 4 at arrival to the recovery ward; IQR: 3–4). Patient satisfaction at discharge from the recovery ward was in the same range. Mean satisfaction score was 4.4 ± 0.6 (median satisfaction score was 5; IQR: 4–5).

### Adverse events

There were six procedural complications (SAEs) in the form of pneumothoraxes (although no drainage was needed, this was considered potentially life-threatening) and five reported post-procedural adverse events, one of which was a perioperative cardiac arrest, which was successfully resuscitated. This SAE was, at least partly, explained by the fact that a high dose of remifentanil was administered shortly before the termination of the intervention. After this incident, our handling routines of extensive pain were somewhat modified, and use of local anaesthetic was further encouraged. The other AEs that required treatments were severe nausea (*n* = 2), pain in shoulder (*n* = 1), and pruritus (*n* = 1).

## Discussion

The possibility to perform therapeutic and/or diagnostic interventions is not limited to interventional radiology; neurosurgery, endoscopy, and vascular surgery are other fields where analgosedation may be favourable both to severely disabled patients as well as from a health-economical perspective. There is no reason to assume that interventional activities will be limited to the present fields. Therefore, it is our intention that the present study will contribute to the narrative of future investigations, aiming to explore new options in the field of sedative comfort without interfering with long-term results. Anaesthetic experience (general anaesthesia versus conscious sedation) during percutaneous ablation of hepatocellular carcinoma has been retrospectively investigated (10). The choice of the anaesthetic technique depended not only on the tumour, but also on patient factors, since this was a cohort with frequent co-morbidities.

Although some patients scored high pain levels during the renal intervention, the overall patient comfort in this prospective investigation seems satisfactory, since there were no significant differences in expected versus experienced periprocedural pain. Also, the need for supplementary morphine during the procedure was 8%, suggesting that the vast majority of patients found the administered amounts of dexmedetomidine and remifentanil sufficient. Postoperative pain was experienced in nearly three out of four patients, which is not surprising due to the fact that short-acting drugs were used. However, the total amount of opioids given at the recovery unit was not high.

Dexmedetomidine is a potent alfa-2 agonist, having anxiolytic, analgesic, and sedative properties. It is an efficient adjunct to regional anaesthesia and reduces the need for opioids. Furthermore, dexmedetomidine may decrease the inflammatory response to renal ischaemia, which may be an advantage in this context (11). The other drug used was remifentanil, with favourable pharmacodynamic properties in this setting, since it is metabolized by esterases, making it minimally altered by age or renal or hepatic dysfunction. Remifentanil has analgesic properties with minimal effect on cognitive function (12). This combination may be advantageous for the purpose of analgosedation, where unconsciousness is not desirable and muscle relaxation is not necessary.

Our use of dexmedetomidine and remifentanil in the roentgenological department is an example of the known demand for sedation during regional anaesthetic procedures outside the operating room (13). In the future we can expect that different methods of applying sedation will be used, since there is sometimes a shortage of qualified anaesthetists, bringing up a discussion whether non-anaesthesiologists can be responsible for qualified sedation. Against the background of this study, such a concept seems questionable, since remifentanil and dexmedetomidine are both examples of potent drugs. These challenges are mastered through the use of well-educated staff familiar with drugs and equipment (e.g. target-controlled infusion technique), to facilitate safety and efficacy during such procedures (14). However, quick access to a physician capable of managing life-threatening events is a must.

Pneumothoraxes may occur during percutaneous ablation of renal carcinoma. Spontaneously breathing patients may be less prone to develop intrapleural inflation of gas, if the visceral pleura is perforated, compared to general anaesthesia, where intermittent positive pressure ventilation is applied, and, especially, when nitrous oxide is used. According to our experience, the incidence of pneumothoraxes in this study is in the same range as in fully anaesthetized patients.

This study has several limitations. The most obvious one is the fact that this was not a randomised controlled trial. In a retrospective study performed between 2010 and 2015, including 51 patients, Kim et al. (15) found that general anaesthesia seems superior to conscious sedation for local tumour control. Although their findings may favour general anaesthesia, it should be remembered that some patients are not suitable for general anaesthesia. Also, the mean age of their patients was 57 years, compared to ours, which was 66 years. Furthermore, their mean treatment time of sedated patients was significantly shorter compared to anaesthetized patients, but in the same range as in our study. Another drawback of our study, as well as the study by Kim et al. (15), is the limited number of patients, as well as the fact that only a small number of roentgenological or anaesthetic staff was involved. Preoperative paracetamol was administered to 40% of the patients, which might have reduced pain perception in some cases. Ketobemidone was administered to two patients suffering from nausea and emesis in an attempt to reduce this discomfort. Also, ours was a single-center study with a heterogeneous population, and our results should, therefore, be interpreted with care from a global aspect.

## Conclusions

Sedation with dexmedetomidine and remifentanil during percutaneous ablation of renal carcinoma appears to be efficacious, although several patients reported high pain scores.

Somewhat surprisingly, the patients assessed the procedure as overall acceptable. Postoperative pain is a frequent problem, especially during the first two hours after the radiological intervention, and prophylactic analgesic regimen should be applied. However, care must be taken to avoid overdosage of opioids in this population. Analgosedation during ablation of renal carcinoma should be performed only by dedicated and well-trained anaesthetic staff.

A large, preferably multi-center, prospective, randomized controlled trial, comparing analgosedation versus general anaesthesia during percutaneous ablation of renal carcinoma focussing on long-term therapeutic effects, safety, patient comfort, optimal dosage, and health economy would be advantageous. A non-opioid premedication is also recommended in order to limit the need for extra analgesics pre- and postoperatively.
